# Coupling Lipid Labeling and Click Chemistry Enables Isolation of Extracellular Vesicles for Noninvasive Detection of Oncogenic Gene Alterations

**DOI:** 10.1002/advs.202105853

**Published:** 2022-03-09

**Authors:** Na Sun, Benjamin V. Tran, Zishan Peng, Jing Wang, Ceng Zhang, Peng Yang, Tiffany X. Zhang, Josephine Widjaja, Ryan Y. Zhang, Wenxi Xia, Alexandra Keir, Jia‐Wei She, Hsiao‐hua Yu, Jing‐Jong Shyue, Hongguang Zhu, Vatche G. Agopian, Renjun Pei, James S. Tomlinson, Jeffrey A Toretsky, Steven J. Jonas, Noah Federman, Shaohua Lu, Hsian‐Rong Tseng, Yazhen Zhu

**Affiliations:** ^1^ California NanoSystems Institute Crump Institute for Molecular Imaging Department of Molecular and Medical Pharmacology University of California, Los Angeles Los Angeles CA 90095 USA; ^2^ Key Laboratory for Nano‐Bio Interface Suzhou Institute of Nano‐Tech and Nano‐Bionics University of Chinese Academy of Sciences Chinese Academy of Sciences Suzhou 215123 P. R. China; ^3^ Department of Surgery University of California, Los Angeles Los Angeles CA 90095 USA; ^4^ Department of Pathology Zhongshan Hospital Fudan University Shanghai 200032 P. R. China; ^5^ Department of Pathology Shanghai Medical College Fudan University Shanghai 200032 P. R. China; ^6^ Department of Pediatrics David Geffen School of Medicine Eli and Edythe Broad Center of Regenerative Medicine and Stem Cell Research and Children's Discovery and Innovation Institute University of California, Los Angeles Los Angeles CA 90095 USA; ^7^ Smart Organic Materials Laboratory Institute of Chemistry Academia Sinica Nankang Taipei 115 Taiwan; ^8^ Research Center for Applied Sciences Academia Sinica Nankang Taipei 115 Taiwan; ^9^ Departments of Oncology and Pediatrics Georgetown University Washington DC 20057 USA; ^10^ California NanoSystems Institute Departments of Chemistry and Biochemistry and of Materials Science and Engineering University of California, Los Angeles Los Angeles CA 90095 USA

**Keywords:** click chemistry, extracellular vesicles, gene alteration quantification, lipid labeling

## Abstract

Well‐preserved molecular cargo in circulating extracellular vesicles (EVs) offers an ideal material for detecting oncogenic gene alterations in cancer patients, providing a noninvasive diagnostic solution for detection of disease status and monitoring treatment response. Therefore, technologies that conveniently isolate EVs with sufficient efficiency are desperately needed. Here, a lipid labeling and click chemistry‐based EV capture platform (“Click Beads”), which is ideal for EV message ribonucleic acid (mRNA) assays due to its efficient, convenient, and rapid purification of EVs, enabling downstream molecular quantification using reverse transcription digital polymerase chain reaction (RT‐dPCR) is described and demonstrated. Ewing sarcoma protein (EWS) gene rearrangements and kirsten rat sarcoma viral oncogene homolog (KRAS) gene mutation status are detected and quantified using EVs isolated by Click Beads and matched with those identified in biopsy specimens from Ewing sarcoma or pancreatic cancer patients. Moreover, the quantification of gene alterations can be used for monitoring treatment responses and disease progression.

## Graphical abstract

This work demonstrates an efficient and rapid extracellular vesicle (EV) capture platform (“Click Beads”), which enables downstream quantification of gene alterations in both Ewing sarcoma and pancreatic cancer using reverse transcription digital polymerase chain reaction (RT‐dPCR). The streamlined workflow that combines Click Bead‐based EV capture and RT‐dPCR exhibits potential clinical utility in disease detection and treatment response monitoring.

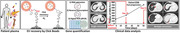

## 1 Introduction

Extracellular vesicles (EVs)^[^ ^1^ ^]^ are a heterogeneous collection of phospholipid bilayer‐enclosed nanoparticles, which are generally classified into the following three subtypes: exosomes (30–100 nm), microvesicles (100–1000 nm), and apoptotic bodies (1000–5000 nm).^[^ ^2^ ^]^ EVs are produced from all cell types and can be found in body fluids and blood circulation, making these nanoparticles one of the three liquid biopsy applications.^[^ ^3^ ^]^ Ribonucleic acid (RNA), deoxyribonucleic acid (DNA), and protein are the biomolecular cargoes encapsulated in EVs, which mirror those in their tissues or organs of origin.^[^ ^4^ ^]^ The detection and characterization of EVs and their cargoes could introduce a rich source of circulating biomarkers for disease diagnosis and treatment monitoring in a noninvasive manner.^[^ ^5^ ^]^ Over the past two decades, a large collection of technologies has been developed to enrich, detect, and analyze EVs.^[^ ^6^ ^]^

Conventional technologies, such as ultracentrifugation,^[^ ^7^ ^]^ filtration,^[^ ^8^ ^]^ precipitation,^[^ ^9^ ^]^ and size‐based microfluidic enrichment,^[^ ^10^ ^]^ were developed to isolate EVs in blood based on their physical properties (i.e., sizes and/or density). However, the ultracentrifugation is not convenient and requires large instrument, and the reported performance of different technologies for EV isolation varies widely.^[^ ^11^ ^]^ Therefore, technologies that conveniently isolate EVs with sufficient efficiency are desperately needed. Novel techniques have been developed for more efficient and convenient EV enrichment methods with the implementation of downstream molecular characterization.^[^ ^12^ ^]^ Zheng et al. developed a powerful EV enrichment technology, which combines lipid labeling and magnetic beads to effectively tag and recover EVs from plasma samples. This allows for downstream analysis of mutations in epidermal growth factor receptor (EGFR) and kirsten rat sarcoma viral oncogene homolog (KRAS) genes in nonsmall‐cell lung‐cancer patients.^[^ ^13^ ^]^ The bilayer membranes are the most prominent characteristics of EVs, and labeling EV membranes by the insertion of lipophilic membrane dyes (e.g., PKH26 and PKH67)^[^ ^14^ ^]^ is the most common way to characterize EVs. The use of lipid labeling allows for EV capture independent of surface antigens, providing the flexibility to capture and enrich all EVs. Building upon this approach, we aim to maximize the utility of lipid‐based labeling for EV capture technologies.

**Figure 1:**
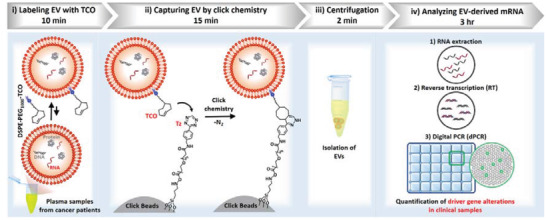
Schematic illustration of a streamlined workflow for capture and characterization of extracellular vesicles (EVs). i) Labeling EV with trans‐ cyclooctene, TCO (10 min): EV in plasma is labeled by TCO via the insertion of the lipid motif of DSPE‐PEG_1000_‐TCO. ii) Capturing EV by click chemistry (15 min): The TCO‐labeled EV is immobilized onto the tetrazine (Tz)‐grafted microbeads (i.e., Click Beads) by a bioorthogonal click chemistry reaction. iii) Rapid centrifugation (2 min): The EVs captured on the Click Beads are isolated via centrifugation at 300 g. iv) Analyzing EV‐derived mRNA by reverse transcription digital PCR (RT‐dPCR): The EVs are first lysed to release EV‐derived mRNA, which is subjected to downstream gene analysis by RT‐dPCR. This workflow is adopted to quantitatively detect gene alterations (i.e., EWS/FLI‐1 rearrangements in Ewing sarcoma and KRAS mutations in pancreatic cancer) in cancer patients.

We introduce a rapid EV enrichment solution that combines the lipid‐based labeling and click chemistry‐mediated EV capture onto microbeads (i.e., Click Beads), thus allowing for isolation of EVs via simple centrifugation. In a streamlined workflow (**Figure** **1**), EVs in plasma are first labeled with trans‐cyclooctene (TCO) via the insertion of the lipid motif of TCO‐tagged 1,2‐distearoyl‐*sn*‐glycero‐3‐phosphoethanolamine‐polyethylene glycol (DSPE‐PEG_1000_‐TCO) into the EV membranes. The DSPE‐PEG_1000_‐TCO conjugate was prepared by incubating TCO‐PEG‐NHS ester with DSPE‐PEG_1000_‐NH_2_, as shown in Scheme S1 (Supporting Information). When adding the tetrazine (Tz)‐grafted microbeads (i.e., Click Beads) into the plasma samples, the TCO‐labeled EVs are immobilized onto Click Beads via a bioorthogonal click chemistry reaction between TCO and Tz motifs. After rapid centrifugation at 300 g for 2 min, the EVs captured onto the Click Beads are collected at the bottom of an Eppendorf tube. The captured EVs are lysed to release EV‐derived message RNA (mRNA), which is then subjected to downstream gene analysis by reverse transcription digital polymerase chain reaction (RT‐dPCR). Previously, click chemistry reactions on the surface of EV Click Chips have demonstrated success in isolating the subpopulation of tumor‐derived EVs in studies focusing on the early detection of hepatocellular carcinoma.^[^ ^15^ ^]^ In this study, Click Beads with DSPE‐PEG_1000_‐TCO enables effective capture of EVs derived from cancer cells of multiple origins, such as epithelial (e.g., pancreatic cancer) and mesenchymal (e.g., Ewing sarcoma) origin, since lipid labeling bypasses the need for surface antigens. We adopted this streamlined workflow to quantitatively detect gene alterations (i.e., Ewing sarcoma protein (EWS) gene rearrangements in Ewing sarcoma^[^ ^16^ ^]^ and KRAS mutations in pancreatic cancer^[^ ^17^ ^]^ patients. We demonstrated that the Click Beads‐based universal capture of total EVs, combined with cancer‐specific gene alteration detection and quantification, allows for noninvasive cancer diagnosis and treatment monitoring.

## 2 Results

### 2.1 Click Beads Modification and Characterization

**Figure 2:**
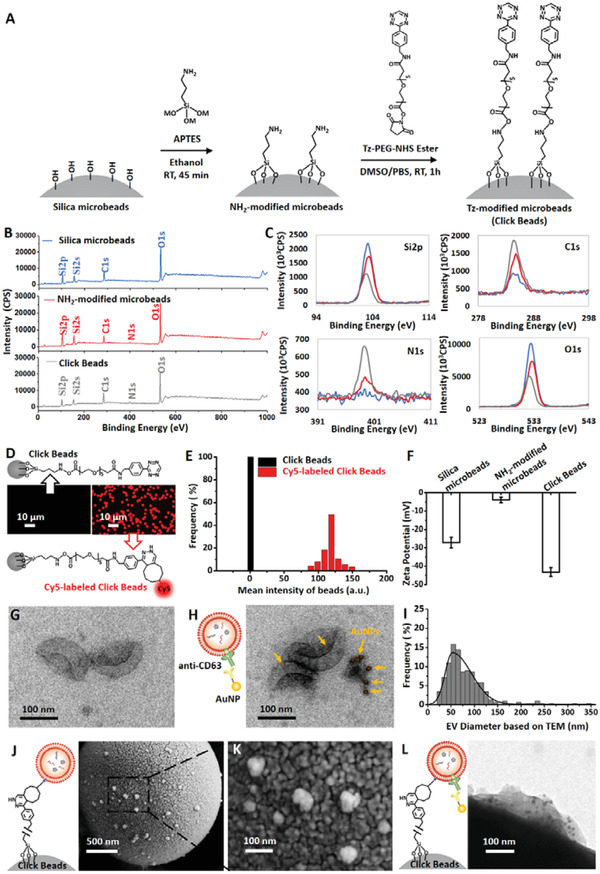
Preparation and characterization of Tz‐grafted microbeads (i.e., Click Beads) before and after EV capture. A) Stepwise preparation of Click Beads. B) Elemental survey of the surface chemical composition of silica microbeads, NH_2_‐modified microbeads and Tz‐grafted microbeads (Click Beads). C) High resolution scan of Si2p, C1s, N1s, and O1s of silica microbeads (blue), NH_2_‐modified microbeads (red), and Tz‐grafted microbeads (Click Beads, gray). D) Fluorescent micrographs of Click Beads before and after labeling by TCO‐conjugated Cy5. E) Histograms of average fluorescence intensity observed for Click Beads and Cy5‐labeled Click Beads. F) The surface zeta potential of silica microbeads, NH_2_‐modified microbeads, and Click Beads. G,H) A representative transmission electron microscopy (TEM) image (scale bar, 100 nm) of EVs derived from A673 cells before and after immunostaining by anti‐CD63‐grafted gold nanoparticles. I) Size distribution (*n* = 291, 30–400 nm in diameter) of A673 cell‐derived EVs, based on TEM imaging. J) A representative scanning electron microscopy (SEM) image (scale bar, 500 nm) of a Click Bead with immobilized EVs. Inset depicts how TCO‐labeled EV is immobilized onto a Click Bead. K) High‐resolution SEM showing the EVs captured on the surface of Click Beads. L) A representative high‐resolution TEM image of EVs captured on a Click Bead after immunostaining by anti‐CD63‐grafted gold nanoparticles.

To enable a rapid workflow for capture and characterization of EVs, we prepared Tz‐grafted microbeads, i.e., Click Beads, which are capable of immobilizing TCO‐labeled EV via a bioorthogonal click chemistry reaction. **Figure** **2**A summarizes the preparation of Click Beads via two major steps. First, 2.5 µm silica microbeads were first modified (3‐aminopropyl) triethoxysilane (APTES) via a silanization reaction to give NH_2_‐modified microbeads. Subsequently, the NH_2_‐modified microbeads are treated with tetrazine‐polyethylene glycol‐*N*‐hydroxysuccinimide (Tz‐PEG‐NHS) ester to yield Click Beads. Surface composition analysis using X‐ray photoelectron spectroscopy (XPS) for Click Beads preparation was shown in Figure 2B,C; and Table S1 (Supporting Information). The obviously increased intensity of the N 1s peak was observed for Click Beads, which is consistent with the higher nitrogen content of the Tz motif. We further tested and verified the preparation of Click Beads via the iorthogonal ligation between Tz on Click Beads and Cy5‐labeled TCO (Figure 2D). The Cy5 fluorophore was grafted onto the Click Beads through a TCO and Tz coupling reaction, leading to a strong fluorescent signal detected on treated Click Beads (Figure 2E). These data demonstrated the efficient grafting of Tz onto the Click Beads and the effectiveness of the click reaction. The surface modification of Click Beads was also monitored by the changes in surface charge, as shown in Figure 2F. APTES was considered to shift the negative surface charge of silica MBs with hydroxyl groups to the positive direction by attaching one amine, resulting in a rise trend of the zeta potential. Then the zeta potential falls due to the formation of amides with the decrease of free amino groups. This shift of surface zeta potential further confirmed the successful preparation of Click Beads with a zeta potential of −43.3 mV.

EVs from A673 cells (an Ewing Sarcoma cell line) were harvested via ultracentrifugation and identified by transmission electron microscopy (TEM), dynamic light scattering (DLS), and nanoparticle tracking analysis (NTA). The EVs imaged with TEM exhibited cup morphologies, as shown in Figure 2G. To confirm the identity of EVs, immunogold staining using anti‐CD63 (an EV surface marker) was employed to label A673 cell‐derived EVs, and 12 nm gold nanoparticles were detected on the surface of EVs (Figure 2H). The diameter distributions of EVs determined via TEM ranged from 30 to 400 nm (Figure 2I), which is consistent with those measured by DLS and NTA revealing a similar distribution from 40 to 500 nm (Figure S1, Supporting Information).

After immobilizing A673 cell‐derived EVs onto Click Beads using the workflow described in Figure 1, scanning electron microscopy (SEM) was employed to characterize the EV/Click Beads interfaces. Following fixation and dehydration, the EVs captured on Click Beads were imaged by SEM (Figure 2J,K), showing a size distribution ranging from 30 to 200 nm (Figure S2, Supporting Information). The inset of Figure 2J includes an illustration of how a TCO‐labeled EV was immobilized onto a Click Bead. The high‐resolution SEM image in Figure 2K shows a nanoscaled rough surfaces of Click Beads, which provides a matching nanostructure for EVs immobilizing onto the beads. The artificial plasma samples spiked with A673 cell‐derived EVs were also used as a model system in this study. The morphology of EVs captured onto Click Beads, imaged by TEM, was shown in Figure S3 (Supporting Information). To further confirm the identity of captured EVs, immunogold staining using anti‐CD63 was also employed. The high‐resolution TEM image in Figure 2L shows that gold nanoparticles were detected on the surface of EVs captured on a Click Bead.

### 2.2 Preparation of Artificial Samples

**Figure 3:**
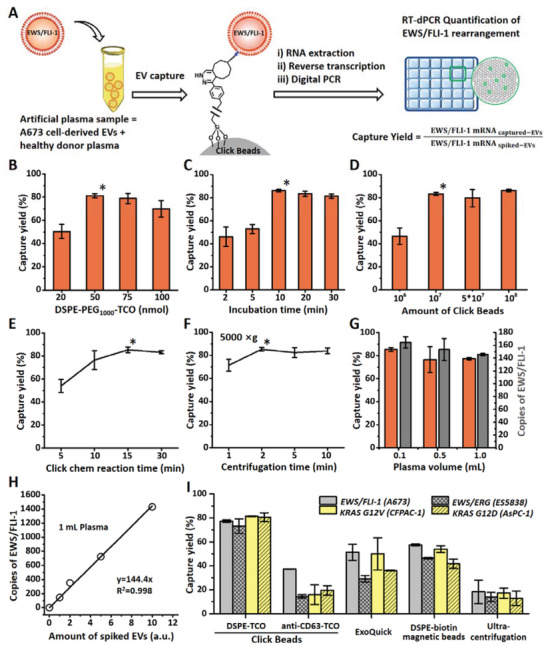
A) A workflow developed for the optimization of EV capture by Click Beads using artificial plasma samples, which contain A673 cell‐derived EVs. EWS/FLI‐1 mRNA was quantified to determine the EV capture yields. EV capture yields as a function of B) amount (nmol) of DSPE‐PEG_1000_‐TCO, C) incubation time (min) of DSPE‐PEG_1000_‐TCO, D) amount of Click Beads, E) click chemistry reaction time (min), and F) centrifugation time (min). G) Data of capture yields and copies of EWS/FLI‐1 mRNA observed for different volumes (mL) of artificial plasma samples by using a fixed ratio of 10^7^ Click Beads to 100 µL of plasma (i.e., 0.1 mL with 10^7^ beads, 0.5 mL with 5 × 10^7^ beads, and 1.0 mL with 10^8^ beads). H) Dynamic range of Click Beads displays a linear correlation between the amount (a.u.) of A673 cell‐derived EVs spiked in 1 mL artificial plasma samples and the detected copies of EWS/FLI‐1 mRNA. I) A comparison of capture yields observed for four types of EV‐spiked artificial plasma samples (two Ewing sarcoma cell lines – A673 and ES‐5838, and two pancreatic cancer cell lines, CFPAC‐1 and AsPC‐1) using the following five EV isolation methods: Click Beads with DSPE‐PEG_1000_‐TCO, Click Beads with anti‐CD63‐TCO, ExoQuick (System Biosciences), magnetic beads with biotin‐conjugated DSPE, and ultracentrifugation.

In order to demonstrate the reproducibility of Click Beads for EV capture throughout the initial optimization process, artificial Ewing sarcoma plasma samples were prepared (**Figure** **3**A) by spiking EVs (10 µL) produced from A673 Ewing sarcoma cells into plasma (90 µL) from a healthy donor (see detailed procedure in the Experimental Section) with a concentration of 1.21 ± 0.04 ×10^10^ particles mL^−1^ (Figure S1, Supporting Information). A673 Ewing sarcoma cells harbor EWS/FLI‐1 rearrangements, which are found in ≈90% of all Ewing sarcoma tumors.^[^ ^18^ ^]^ Since the EWS/FLI‐1 rearrangements are absent in healthy donor's plasma, A673 cell‐derived EVs can be specifically quantified by detecting EWS/FLI‐1 mRNA using RT‐dPCR.

### 2.3 Multifaceted Optimization of Click Beads for EV Capture

An RT‐dPCR assay in Figure 3A was used to quantify the copies of EWS/FLI‐1 mRNA in the artificial Ewing sarcoma plasma samples before and after EV capture by Click Beads. The results can be used to calculate the capture yield throughout the optimization process. We denoted the copy numbers of EWS/FLI‐1 mRNA in the original 10 µL aliquoted A673 Ewing sarcoma EVs and Click Bead‐captured A673 EVs as EWS/FLI‐1 mRNA _spiked‐EVs_ and EWS/FLI‐1 mRNA _captured‐EVs_, respectively. The EV capture yield obtained by Click Beads under a given condition can be calculated using Equation (1) shown in Figure 3A

(1) $$\text{Capture}\;\text{yield}=\frac{\mathrm{EWS}/\mathrm{FLI}-1\;\text{mRN}A_{\mathrm{captured}-\mathrm{EVs}}}{\mathrm{EWS}/\mathrm{FLI}-1\;\text{mRN}A_{\mathrm{spiked}-\mathrm{EVs}}}$$

To optimize the EV capture performance of Click Beads using artificial Ewing sarcoma plasma samples, we examined different experimental parameters, including the amount of DSPE‐PEG_1000_‐TCO, incubation time of DSPE‐PEG_1000_‐TCO in the artificial samples, amount of Click Beads, click chemistry reaction time, and centrifugation time. In each study, 100 µL artificial Ewing sarcoma plasma sample was used. To determine the optimal amount of DSPE‐PEG_1000_‐TCO required for EV capture, various concentrations (20, 50, 75, and 100 nmol) were tested. 50 nmol was found to be the optimal concentration (Figure 3B). Using this amount of DSPE‐PEG_1000_‐TCO, different incubation times ranging from 2 to 30 min were examined. As shown in Figure 1, 3 min was identified as the optimal incubation time. Using 50 nmol DSPE‐PEG_1000_‐TCO at a 10 min incubation time, we next captured EVs using different numbers (10^6^ to 10^8^) of Click Beads. Once the amount of Click Beads exceeded 10^7^ per study, there was no significant difference in the observed capture yields (Figure 3D). We therefore determined 10^7^ Click Beads per sample is sufficient to achieve maximum EV capture yields. The click chemistry reaction time and centrifugation time were also optimized to attain maximum EV capture yields. As shown in Figure 3E,F, optimal click chemistry and centrifugation time are 15 and 2 min, respectively.

Under the optimized EV capture condition described above for 100 µL of plasma, we examined how plasma volumes impact EV capture performance. With a fixed amount of A673 Ewing sarcoma EVs in three different volumes (i.e., 0.1, 0.5, and 1.0 mL) of plasma samples, we demonstrated that EV capture performance (Figure 3G) was not significantly affected by the plasma volume. Given that background EVs increase proportionally with plasma volumes, we introduced a fixed ratio of Click Beads to plasma volume (10^7^ to 100 µL). We then checked the dynamic range of Click Beads using 1.0 mL artificial plasma samples spiked with different concentrations of EVs containing 0–1600 copies of EWS/FLI‐1 mRNA and confirmed the consistency of EV capture yields (*y* = 144.4x, *R* ^2^ = 0.998) (Figure 3H). Together, these results established that the final optimized conditions allow for practical testing of 1.0 mL plasma samples.

To confirm the reproducibility and general applicability of Click Beads with DSPE‐PEG_1000_‐TCO for capturing EVs from different types of tumors, a study was conducted to compare capture yields of this method with those of four other methods: Click Beads with anti‐CD63‐TCO, ExoQuick (System Biosciences), magnetic beads (Dynabeads) with biotin‐conjugated DSPE, and ultracentrifugation. In this study, four types of artificial plasma samples (1.0 mL per study), two spiked with EVs from Ewing Sarcoma cell lines (A673 and ES‐5838, respectively), and the other two were spiked with EVs from pancreatic cancer cell lines (CFPAC‐1 and AsPC‐1, respectively), were prepared and used. For ES‐5838 EVs (with EWS/ERG rearrangement), CFPAC‐1 EVs (with KRAS G12V mutation), and AsPC‐1 EVs (with KRAS G12D mutation), the respective RT‐dPCR assays were developed to quantify the spiked and captured EVs. We then calculated the respective EV capture yields by adopting formulas (see the Supporting Information) similar to Equation (1). As shown in Figure 3I, Click Beads with DSPE‐PEG_1000_‐TCO exhibited superior capture yields (ranging from 73.1% ± 6.0% to 80.6% ± 3.6%) for all four cell line‐derived EVs in comparison to capture yields of Click Beads with anti‐CD63‐TCO (ranging from 14.5% ± 1.4% to 37.2% ± 0.1%). The latter technique exhibited consistently low capture performance due to the dependence of CD63 on EV surfaces. Meanwhile, the capture yields of the three control studies, including ExoQuick ULTRA EV Isolation Kit (ranging from 29.1% ± 2.7% to 51.2% ± 6.7%), magnetic beads using biotin‐PEG‐DSPE conjugates (ranging from 41.8% ± 3.9% to 57.5% ± 0.9%), and ultracentrifugation (ranging from 12.8% ± 6.1% to 18.2% ± 9.7%), were all lower than those of Click Beads with DSPE‐PEG_1000_‐TCO. Overall, these results further verified the reproducibility and general applicability of Click Beads with DSPE‐PEG_1000_‐TCO for capturing EVs, providing a framework for future studies using clinical samples.

### 2.4 Quantification of EWS Rearrangements in EVs Captured by Click Beads from Ewing Sarcoma Patients

**Figure 4:**
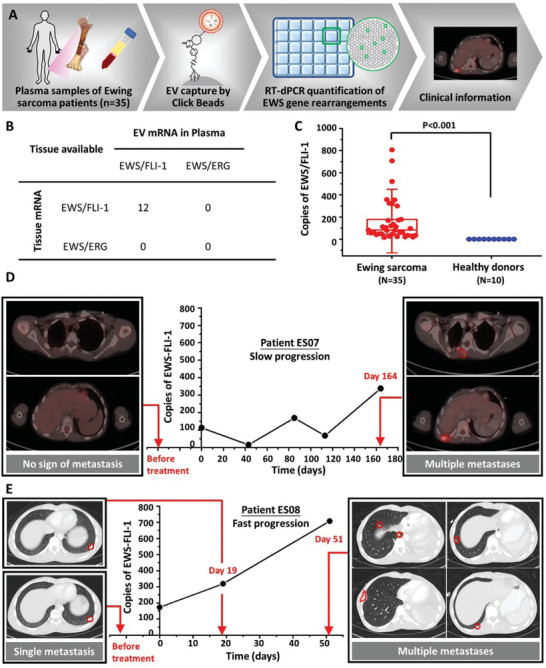
A) The workflow (Click Bead‐based EV capture + RT‐dPCR quantification) developed for the quantification of EWS gene rearrangements in Ewing sarcoma patients’ plasma samples. B) The comparison of two subtypes of EWS rearrangements (i.e., EWS/FLI‐1 and EWS/ERG) of paired tissue and plasma samples from 12 Ewing sarcoma patients. C) Box plots of the EWS/FLI‐1 mRNA copies detected for Ewing sarcoma patients (*N* = 35) and healthy donor samples (*N* = 10). Whiskers ranging from minima to maxima, median and 25–75% IQR shown by box plots. Significant differences between different groups were evaluated using *t*‐test (*P* < 0.001). D) A plot of copies of EWS/FLI‐1 mRNA (per 1.0 mL blood) versus times of the assay depicting the slow disease progression of an Ewing sarcoma patient (ES07). Positron Emission Tomography – Computed Tomography (PET/CT) images taken before and 164 days after treatment. E) A plot of copies of EWS/FLI‐1 mRNA versus times depicting the fast disease progression of an Ewing sarcoma patient (ES08). CT images taken before, at 19 days, and 51 days post‐treatment.

Following the previously described optimization of the Click Beads with DSPE‐PEG_1000_‐TCO protocol, we then explored its clinical utility in Ewing sarcoma with an modified workflow to quantify the two most common subtypes of EWS gene rearrangements (i.e., EWS/FLI‐1 and EWS/ERG, Figure S4 and Table S2, Supporting Information) in Ewing sarcoma patients (**Figure** **4**A). In total, 35 plasma samples from 28 patients with clinically confirmed Ewing sarcoma and 10 plasma samples from 10 healthy donors were analyzed (Tables S3 and S4, Supporting Information). Each study required 1 mL of plasma per sample. Among the 28 Ewing sarcoma patients, the EWS/FLI‐1 and EWS/ERG rearrangements were compared between plasma samples and the respectively paired undecalcified formalin‐fixed paraffin‐embedded (FFPE) tissue samples of 12 Ewing sarcoma patients. The results summarized in Figure 4B showed that all 12 patients harbor EWS/FLI‐1 rearrangements in EVs captured from plasma samples, consistent with the results from their respectively paired biopsies obtained at diagnosis. Figure 4C showed that EWS rearrangements were detected in the EVs captured from all Ewing sarcoma plasma samples, but not detected in the EVs captured from healthy donor plasma samples. Next, we aligned the copies of EWS/FLI‐1 mRNA (obtained by following the optimized workflow) with Positron Emission Tomography – Computed Tomography (PET/CT) images in order to monitor the dynamic changes in Ewing sarcoma in relation to treatment response. This was performed on 2 patients with serial samples taken before the initiation of treatment and up to164 days post‐treatment. Serial plasma samples were collected from patient ES07 with confirmed EWS/FLI‐1 rearrangement before treatment, and at 43, 85, 113, and 164 days post‐treatment. The corresponding copies of rearrangements for each timepoint were plotted in Figure 4D. PET/CT images of the patient showed that prior to treatment, the patient exhibited no sign of tumor metastasis. Following the initiation of chemotherapy, the patient was found to have a partial response after 43 days of treatment but subsequently relapsed after at 164 days post‐treatment with multiple metastases. These radiographic observations are consistent with the dynamic changes observed in the copies of EWS/FLI‐1 mRNA. Similarly, serial plasma samples were collected from patient ES08 with confirmed EWS/FLI‐1 rearrangement before treatment, and 19 and 51 days post‐treatment. Figure 4E showed that CT images before treatment, and at 19 and 51 days post‐treatment delineated aggressive progression from a single metastatic depot to multiple metastases, which strongly correlated with the increase of the copies of EWS/FLI‐1 mRNA.

### 2.5 Quantification of KRAS Mutations in EVs Captured by Click Beads from Pancreatic Cancer Patients

**Figure 5:**
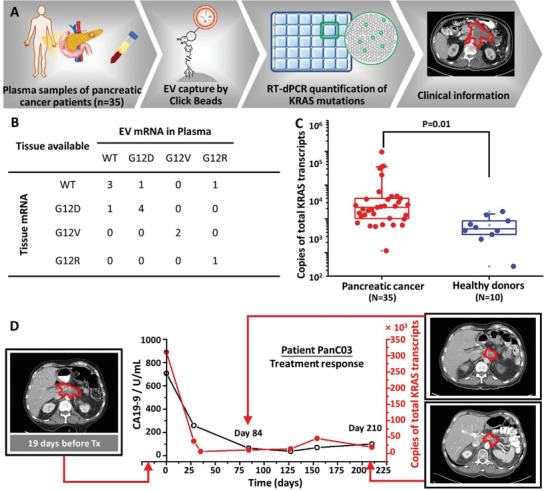
A) The workflow (Click Bead‐based EV capture + RT‐dPCR quantification) developed for quantification of KRAS mutations in pancreatic cancer patients’ plasma samples. B) The comparison of KRAS wild type and three subtypes of KRAS mutations transcripts (i.e., G12D, G12V, G12R) of paired tissue and plasma samples from 13 pancreatic cancer patients. C) Box plots of the total KRAS transcript copies detected for pancreatic cancer patients (*N* = 35) and healthy donor samples (*N* = 10). Whiskers ranging from minima to maxima, median and 25–75% IQR shown by box plots. Significant differences between different groups were evaluated using *t*‐test (*P* = 0.01). D) A plot of copies of total KRAS mutation transcripts (per 1.0 mL blood) and CA19‐9 levels (U mL^−1^) versus times of the assay depicts the treatment response of a pancreatic cancer patient (PanC03). Computed Tomography (CT) images taken 19 days before, 84 days, and 210 days after treatment.

We tested the clinical utility of the modified workflow (**Figure** **5**A) in pancreatic cancer as its epithelial cellular origin differs from the mesenchymal cellular origin of Ewing sarcoma, broadening the clinical applicability of this technique. In pancreatic adenocarcinoma, the three most common subtypes of KRAS mutations mRNA (i.e., G12D, G12V, and G12R, Figure S5 and Table S5, Supporting Information) were examined. In total, 35 plasma samples from 19 patients with clinically confirmed pancreatic cancer and 10 plasma samples from 10 healthy donors (Tables S4 and S6, Supporting Information) were analyzed. Each study required 1 mL of plasma per sample. Among the 19 pancreatic cancer patients, 13 of them have paired FFPE tissue samples. We then compared the three KRAS mutation status in paired FFPE tissue and plasma samples from 13 patients. The results summarized in Figure 5B showed that matched KRAS mutation subtypes were detected in EVs captured from plasma samples and FFPE tissues obtained at diagnosis in 10 of the 13 pancreatic cancer patients. Among the remaining 3 pancreatic cancer patients with discordant KRAS mutation status, two patients had the KRAS mutations (1 G12D, 1 G12R) detected in plasma samples but not detected in tissue samples, which may result from the tissue heterogeneity. One patient had the KRAS G12D mutation detected in tissue sample but not in the plasma sample presumably due to the copy number change of KRAS mutation over the period between tissue biopsy and blood draw. Figure 5C showed that total copies of KRAS transcripts (mutated KRAS + wild‐type KRAS) were significantly higher in the EVs captured from plasma samples of pancreatic cancer patients compared to the total copies of KRAS transcripts in EVs captured from plasma samples of healthy donors (*P* = 0.01). No mutated KRAS was detected in the plasma samples of healthy donors. We monitored the dynamic changes in one pancreatic cancer patients (PanC03) during treatment by aligning the total copies of KRAS transcripts (obtained by following the modified workflow) and serum CA19‐9 levels (U mL^−1^) with CT images taken before and post‐treatment. Serial plasma samples were collected from patient PanC03 19 days prior to initiation of treatment and 84 and 210 days post‐treatment. The corresponding serum CA19‐9 levels and copies of KRAS transcripts for each timepoint were plotted in Figure 5D. At the final serial sample timepoint (210 days post‐treatment), the patient's treatment response was durable. The radiographic observations were consistent with the dynamic changes observed in both the total KRAS transcripts detected in EVs captured from serial plasma samples and the matched serum CA19‐9 levels.

## 3 Conclusion

In this study, a rapid EV enrichment technology that combines the lipid‐based EV labeling using DSPE‐PEG_1000_‐TCO and click chemistry‐mediated EV capture onto Click Beads has been demonstrated to enable downstream molecular analysis of EVs. After conducting multifaceted optimization of the experimental conditions, we established a streamlined workflow which integrates Click Bead‐based EV capture and RT‐dPCR quantification of EV‐derived mRNA. This workflow was adopted to quantitatively detect gene alterations, i.e., EWS rearrangements in Ewing sarcoma and total KRAS transcripts including both mutated KRAS and wild‐type KRAS in pancreatic cancer, allowing for noninvasive cancer diagnosis and treatment monitoring.

In contrast to immunoaffinity‐based EV labeling which is constrained by the limited numbers of EV‐specific surface antigens (e.g., CD63, CD9, and CD81) on the EV membranes,^[^ ^19^ ^]^ the lipid‐based EV labeling by DSPE‐PEG_1000_‐TCO is independent of EV surface antigens. The insertion of the lipid motif of DSPE‐PEG_1000_‐TCO effectively and specifically grafts TCO onto EVs. In the presence of Click Beads, the TCO‐labeled EVs are instantaneously immobilized onto Click Beads via a bioorthogonal and irreversible click chemistry reaction between TCO and Tz motifs.^[^ ^20^ ^]^ Through rapid and simple centrifugation, the EVs captured on the Click Beads are collected. Overall, Click Beads with DSPE‐PEG_1000_‐TCO uniquely leverages the convenience of lipid‐based EV labeling and specificity and efficiency of click chemistry, introducing an effective EV capture method that preserves biomolecular cargoes (i.e., mRNA) within the EVs for further analysis.

The EV‐derived mRNA obtained from Click Bead‐based EV capture is of high quality and can undergo downstream RT‐dPCR assay. One application is the detection and quantification of EWS rearrangements in patients with Ewing sarcoma, an aggressive bone and soft‐tissue tumor that occurs primarily in the adolescent and young adult population.^[^ ^21^ ^]^ This technological development has the potential to advance the standard of care of this disease, as bone tissue biopsies utilized in the routine pathological diagnosis of this disease undergo decalcification when preparing FFPE slides, which severely damages the quality of the mRNA available for downstream EWS genotyping, rendering it infeasible for mRNA detection in decalcified tissues.^[^ ^22^ ^]^ This clinical challenge is highlighted by the fact that out of 28 Ewing sarcoma patients, only 12 had satisfactory FFPE tissues from metastatic non‐bone tumor deposits that were able to undergo EWS rearrangement confirmation using RT‐dPCR. After the initial diagnosis, dynamic monitoring of clinical treatment response is needed. The Click Bead‐based EV capture method combined with RT‐dPCR provides a noninvasive diagnostic solution, offering a treatment‐response monitoring for Ewing sarcoma patients.

Furthermore, we demonstrated the general applicability of Click‐Bead based EV capture method by expanding its clinical utility to pancreatic cancer. Here, three commonly‐seen KRAS mutations (i.e., G12D, G12V, and G12R)^[^ ^17b^ ^]^ were detected in majority of the pancreatic cancer patients and correlated with KRAS mutation status of the matched tissues. Three patients showed inconsistent KRAS status in tissue and paired plasma samples, and this probably could be attributed to the heterogeneity of the single biopsy tissue^[^ ^23^ ^]^ or the copy number changes of mutated KRAS over the period between tissue biopsy and blood draw. Wild‐type KRAS were detected for all patients, and the higher copy numbers of total KRAS transcripts were used to differentiate pancreatic cancer from healthy donor and monitor the disease progression of pancreatic cancer.

Overall, EVs captured by our Click Beads can be a potential noninvasive diagnostic tool for informing upfront treatment response or therapeutic effectiveness of salvage therapies, and relapse/recurrence monitoring for those cancer patients in remission. Although current and emerging techniques of radiologic imaging (CT chest, abdomen, pelvis, and/or MRI primary site) have been used to detect tumor recurrence and evaluate treatment response.^[^ ^24^ ^]^ Recently, it has been reported that quantification of circulating EVs can be used for monitoring therapy efficiency in cancer.^[^ ^25^ ^]^ EVs can be a potential noninvasive diagnostic solution for detecting cancer of either epithelial origin (e.g., pancreatic adenocarcinoma) or mesenchymal origin (e.g., Ewing sarcoma).

In conclusion, coupling lipid labeling and click chemistry enables effective isolation of EVs for noninvasive detection, characterization, and quantification of oncogenic gene alterations in both Ewing sarcoma and pancreatic cancer. Our streamlined workflow that combines Click Bead‐based EV capture and RT‐dPCR‐based quantification of oncogenic gene alterations showed potential clinical applications in detecting disease progression and monitoring treatment responses. Compared to our previously published EV purification method by EV Click Chips,^[^ ^15^ ^]^ Click Beads can be adopted in most of the research laboratories without specialized device setting to handle a broader range of volumes of plasma samples. We also note that our current study does have some limitations, for example limited cohorts for the both tissue and plasma samples. Testing such a streamlined workflow using study cohorts with larger sample sizes will be implemented in our future plan.

## 4 Experimental Section

### Collection of EVs from Cell Culture Supernatant

Ewing sarcoma cell lines of A673, ES5838 or pancreatic cancer cell lines of CFPAC‐1, AsPC‐1 was cultured in 18 dishes (Thermo Scientific Nunc EasYDish Dishes) under usual conditions until 70–80% confluency. Next, cells were cultured with exosome‐production medium (13 mL per dish) for 24 h. A total of 228 mL conditional medium was collected and centrifugated at 300 g, 4 °C for 10 min followed by another centrifugation step at 2800 g, 4 °C for 10 min to discard cell debris. The medium was carefully transferred to Ultra‐Clear Tubes (38.5 mL, Beckman Coulter, Inc., USA) and then ultracentrifuged at 100 000 g, 4 °C for 90 min. The captured EVs were suspended in 200 µL PBS as original EV samples. For the artificial plasma samples, each 10 µL aliquot of EV pellets was spiked into 90 µL healthy donors’ plasma.

### Fabrication of Tetrazine‐Grafted Silica Microbeads (Click Beads)

The Click Beads (10 mg, 6 × 10^8^ beads, 2.5 µm in diameter and 2.0 g cm^−3^ of density) were first washed through an acid incubation (2.0 N HNO_3_, 10 min) for the regeneration of hydroxyl groups, and followed by immediate silanization in an ethanol solution (600 µL) containing 4% (3‐aminopropyl)triethoxysilane (APTES, 25 µL) for 45 min at room temperature. The amino‐functionalized silica microbeads were washed with ethanol three times to remove the unbound silane and then reacted with Tetrazine‐PEG‐NHS ester (0.94 mg, 3.8 × 10^−3^ m) in DMSO/PBS (PH = 9.0, 600 µL) for 1.0 hr.

Cy5‐labeled TCO reagent (5 × 10^−3^ m) in PBS (100 µL) was used to treat Click Beads (100 µL, 1 × 10^8^ beads) for 30 min. Prior to use, the Click Beads were rinsed with deionized water five times to remove any unbound Cy5‐labeled TCO. Fluorescence images were collected with a Nikon 90i fluorescence microscope (*λ*_ex_ = 590–650 nm, exposure time = 500 ms).

### EV Capture from Plasma Samples

A solution of DSPE‐PEG_1000_‐TCO conjugate was prepared by incubating TCO‐PEG‐NHS ester (5.0 × 10^−3^ m) with DSPE‐PEG_1000_‐NH_2_ (5.0 × 10^−3^ m) in a DMSO/PBS solution at room temperature for 30 min. The DSPE‐PEG_1000_‐TCO solution was stored at −20 °C until use.

10 µL of original EVs were added to 90 µL (or 500 µL, 1mL) of plasma (collected from a healthy donor) to prepare artificial plasma samples, and the amount of EVs spiked into each sample was standardized by RT‐dPCR. Then 10, 50, 75, 150 nmol of DSPE‐PEG_1000_‐TCO were mixed with artificial plasma samples for 5–20 min at room temperature, and TCO‐grafted EVs plasma samples were obtained.

The plasma samples containing TCO‐grafted EVs were incubated with Click Beads for 5–30 min followed by a centrifugation at 5000 g for 2 min. In different EV isolation methods testing, the respective optimal capture conditions were applied for Click Beads (DSPE‐TCO and anti‐CD63‐TCO (50 ng/100 µL) and DSPE‐biotin magnetic beads. Additionally, 100 µL of artificial plasma sample with the same amount of original EVs was added to 38 mL PBS and ultracentrifuged once at 100 000 g and 4 °C for 90 min. 100 µL of artificial plasma samples with the same amount of original EVs were used as the input for ExoQuick tests, the EVs were purified according to the manufacturer's protocol.

### Characterization of EVs

For SEM, EVs captured onto Click Beads were fixed in 4% paraformaldehyde (PFA, in PBS) for 1 h. The samples were then sequentially immersed in 30%, 50%, 75%, 85%, 95%, and 100% (twice) ethanol solutions for 10 min per solution, followed by sputter‐coating with gold at room temperature. The morphology of the EVs was examined using a ZEISS Supra 40VP scanning electron microscope.

For TEM, ultracentrifuged EV samples were fixed in 4% PFA (in PBS) for 30 min. Then the samples were dropped on a 400‐mesh carbon‐coated copper grid and incubated for 10 min at room temperature. Excess samples were blotted with filter paper and washed 5 times by water. Grids were dried for TEM imaging by a T20 iCorr (FEI) high‐resolution cryo‐TEM at an accelerating voltage of 80 kV. For immunogold staining, fixed EVs in PBS or captured on Click Beads were incubated with anti‐CD63 (Abcam, mouse, 1:100 dilution) for 30 min. Then, these samples were incubated with antimouse nanogold (12 nm, 1:50 dilution) for 1 h. The gold‐labeled samples were dropped onto carbon coated copper grids and incubated for 10 min before being wiped off from the grids. After being rinsed 5 times using water, grids were then dried for TEM imaging. The ultra‐centrifuged EV samples (10 µL) were diluted into 1 mL in PBS for DLS and NTA tests.

### RNA Extraction and RT‐dPCR Analysis

The captured EVs on Click Beads were lysed using a QIAzol lysis reagent. A miRNeasy mini kit (Qiagen, Germany) was used to extract and purify RNA from the isolated EVs. The RNA concentration in the EVs was measured using a Qubit 3.0 Fluorometer (Thermo Fisher Scientific, USA) in combination with the Qubit RNA HS Assay (Thermo Fisher Scientific, USA) according to the manufacturer's protocol.

The collected RNA (9.4 µL) was reversely transcribed to complementary DNA (cDNA, 15 µL) using a Thermo Scientific Maxima H Minus Reverse Transcriptase Kit (Thermo Fisher Scientific, USA). Complementary DNA was synthesized under the condition of 55 °C for 30 min and 85 °C for 5 min, and then preamplification was performed using 4.0 µL of cDNA as input to obtain 10 µL preamplified product for each gene according to the manufacturer's instructions of Prelude PreAmp Master Mix Kit (Takara Bio).

For dPCR, the reaction mixture (40 µL) including 4 µL of preamplified product was loaded into each well of a nanoplate (26 K, 24 wells). The nanoplate was transferred into the QIAcuity instrument (Qiagen, Germany) for the following PCR process. A programmed Thermal Cycler was set at 95 °C for 2 min followed by 40 cycles of 95 °C for 15 s and 60 °C for 30 s. The readouts of positive and negative partitions were counted automatically by the instrument and analyzed via QIAcuity software.

### Clinical Blood Sample Processing

Peripheral venous blood samples were collected from patients or healthy donors with written informed consent according to the institutional review board protocols (IRB #14‐000 197) at UCLA. Each 10 mL blood sample was collected in a BD Vacutainer plastic tube (BD, Cat. #366 643) with EDTA. Samples were processed according to the manufacturer's protocol within 4.0 h of collection. The final plasma samples were collected after centrifugation at 10 000 g for 10 min and aliquoted and stored in ‐80 °C refrigerators. Each plasma samples were centrifuged at 10 000 g for 10 min after thawing at 37 °C. 1.0 mL plasma samples were then incubated with DSPE‐PEG_1000_‐TCO at room temperature for 10 min before incubation with Click Beads for EV capture.

### Analysis on Ewing Sarcoma and Pancreatic Cancer Tissue Sections

Serial 4 µm thick tissue sections from FFPE blocks were cut and mounted on poly‐*L*‐lysine coated glass slides. EWS rearrangements analysis of tissues from Ewing sarcoma patients and KRAS mutation analysis of tissues from pancreatic cancer patients were performed. Total RNA was extracted from FFPE tissue sections using Qiagen RNeasy FFPE kit (Qiagen, Germany), followed by RT‐dPCR.

## Conflict of Interest

Following the management plan provide by UCLA Conflict of Interest Review Committee, Dr. Hsian‐Rong Tseng would like to disclose that (1) he has a financial interest in CytoLumina Technologies Corp. and Pulsar Therapeutics Corp., and (2) the UC Regents have licensed intellectual properties invented by Dr. Tseng to CytoLumina and Pulsar. Other authors declare no conflict of interest.

## Author Contributions

N.S., Y.Z., and H.R.T. designed and performed most of the research and data analysis. N.S. performed the preparation and optimization of Click Beads. N.S. and P.Y. performed DLS, Zeta potential, SEM and TEM tests. J.S., H.Y., J.S. performed XPS analysis. B.T. and Z.P. performed the collection of blood samples and clinical information with assistance from S.J., J.T., N.F., and S.L. N.S., Z.P., J.W., C.Z., and R.Z. performed blood sample tests. N.S. and C.Z. performed FFPE sample testing. N.S. and Y.Z. made schematic diagrams and figures with assistance from T.Z. W. X., T.Z., J.W., N.S., Y.Z., and H.R.T. wrote and revised the manuscript with input from A.K., H.Z., V.A., R.P., J.T., J.T., S.J., and N.F. Y.Z. and H.R.T. oversaw project execution.

## Supporting information

Supporting Information

## Data Availability

The data that support the findings of this study are available from the corresponding author upon reasonable request.
