# Supplementary material for: Coupling Lipid Labeling and Click Chemistry Enables Isolation of Extracellular Vesicles for Noninvasive Detection of Oncogenic Gene Alterations
Source: Adv Sci (Weinh). 2022 Mar 9;9(14):2105853. doi: 10.1002/advs.202105853 (PMC9108594; doi:10.1002/advs.202105853)
Supplement: Supplementary file 1 — Supporting Information [file ADVS-9-2105853-s001.pdf]

ADVANCED SCIENCE
Open Access

Supporting Information

for *Adv. Sci.,* DOI: 10.1002/advs.202105853

Coupling Lipid Labeling and Click Chemistry Enables Isolation of Extracellular Vesicles for Noninvasive Detection of Oncogenic Gene Alterations

Na Sun, Benjamin V. Tran, Zishan Peng, Jing Wang, Ceng Zhang, Peng Yang, Tiffany X. Zhang, Josephine Widjaja, Ryan Y. Zhang, Wenxi Xia, Alexandra Keir, Jia-Wei She, Hsiao-hua Yu, Jing-Jong Shyue, Hongguang Zhu, Vatche G. Agopian, Renjun Pei, James S. Tomlinson, Jeffrey A Toretsky, Steven J. Jonas, Noah Federman, Shaohua Lu,* Hsian-Rong Tseng,* and Yazhen Zhu**

[1]California NanoSystems Institute, Crump Institute for Molecular Imaging, Department of Molecular and Medical Pharmacology, University of California, Los Angeles, Los Angeles, CA 90095, USA.

[2]Key Laboratory for Nano-Bio Interface, Suzhou Institute of Nano-Tech and Nano-Bionics, University of Chinese Academy of Sciences, Chinese Academy of Sciences, Suzhou 215123, P.R. China.

[3]Department of Surgery, University of California, Los Angeles, Los Angeles, CA 90095, USA.

[4]Department of Pathology, Zhongshan Hospital, Fudan University, Shanghai 200032, P.R. China.

[5]Department of Pathology, Shanghai Medical College, Fudan University, Shanghai 200032, P.R. China.

[6]Department of Pediatrics, David Geffen School of Medicine, Eli and Edythe Broad Center of Regenerative Medicine and Stem Cell Research, and Children's Discovery and Innovation Institute, University of California, Los Angeles, Los Angeles, CA 90095, USA.

[7]Smart Organic Materials Laboratory, Institute of Chemistry, Academia Sinica, Nankang, Taipei 115, Taiwan

[8]Research Center for Applied Sciences, Academia Sinica, Nankang, Taipei 115, Taiwan

[9]Departments of Oncology and Pediatrics, Georgetown University, Washington DC 20057, USA.

[10]California NanoSystems Institute, Departments of Chemistry and Biochemistry and of Materials Science and Engineering, University of California, Los Angeles, Los Angeles, CA 90095, USA.

*To whom correspondence should be addressed. e-mail: hrtseng@mednet.ucla.edu (H.-R.T.); lushaohua2010@126.com (S.L.); rjpei2011@sinano.ac.cn (R.P.); yazhenzhu@mednet.ucla.edu (Y.Z.).

List

Scheme S1. Conjugation of DSPE-PEG$_{1000}$-TCO..

Figure S1. Size distributions of A673 EVs measured by (A) dynamic light scattering (DLS) and (B) nanoparticle tracking analysis (NTA).

Figure S2. Size distribution of A673 cell-derived EVs on Click Beads based on SEM imaging.

Figure S3. A representative TEM image of EVs captured on a Click Bead.

Figure S4. Schematic diagram of EWS rearrangements (EWS/FLI-1 and EWS/ERG).

Figure S5. Schematic diagram of KRAS mutations (G12D, G12V and G12R).

Table S1. The atomic concentration (relative) obtained from the integration of high-resolution spectrum after Shirley background subtraction.

Table S2. Primers and probes for the detection of EWS rearrangements.

Table S3. Clinical characteristics and plasma EV-RNA based detection of EWS rearrangements of sarcoma patients

Table S4. Clinical characteristics and plasma EV-RNA based detection of healthy donors

Table S5. Primers and probes for the detection of KRAS mutations.

Table S6. Clinical characteristics and plasma EV-RNA based detection of KRAS mutation of pancreatic patients

Supporting Information

For ES-5838 EVs (with *EWS/ERG* rearrangement), CFPAC-1 EVs (with *KRAS G12V* mutation), and AsPC-1 EVs (with *KRAS G12D* mutation), the respective RT-dPCR assays were developed to quantify spiked and captured EVs. We calculate the respective EV capture yields by adopting formulas as following.

ES-5838 EVs

$$\text{Capture yield} = \frac{\text{EWS/ERG mRNA}_{\text{captured}-\text{EVs}}}{\text{EWS/ERG mRNA}_{\text{spiked}-\text{EVs}}} \qquad (\text{S1})$$

CFPAC-1 EVs

$$\text{Capture yield} = \frac{\text{G12V mRNA}_{\text{captured}-\text{EVs}}}{\text{G12V mRNA}_{\text{spiked}-\text{EVs}}} \qquad (\text{S2})$$

AsPC-1 EVs

$$\text{Capture yield} = \frac{\text{G12D mRNA}_{\text{captured}-\text{EVs}}}{\text{G12D mRNA}_{\text{spiked}-\text{EVs}}} \qquad (\text{S3})$$

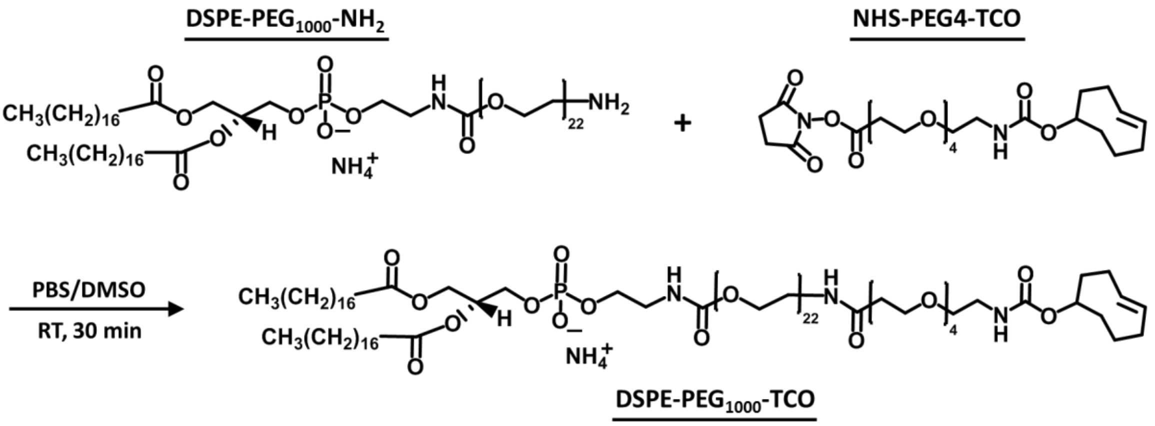

Scheme S1. Conjugation of DSPE-PEG$_{1000}$-TCO. A solution of DSPE-PEG$_{1000}$-TCO conjugate was prepared by incubating TCO-PEG4-NHS ester (5 mM) with DSPE-PEG$_{1000}$-NH$_2$ (5 mM) in a DMSO/PBS (v/v, 1/1) solution at room temperature for 30 min. The DSPE-PEG$_{1000}$-TCO solution was stored at -20 °C until use.

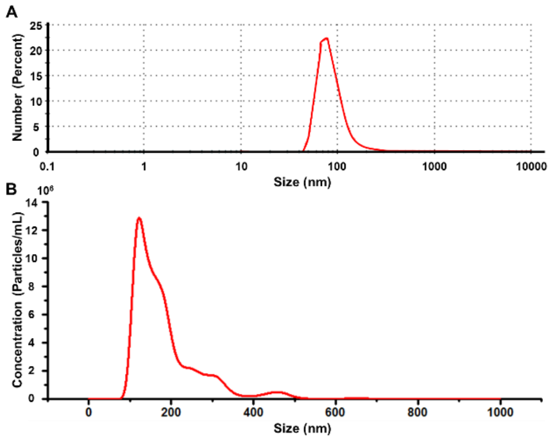

Figure S1. Size distributions of A673 EVs measured by (A) dynamic light scattering (DLS) and (B) nanoparticle tracking analysis (NTA).

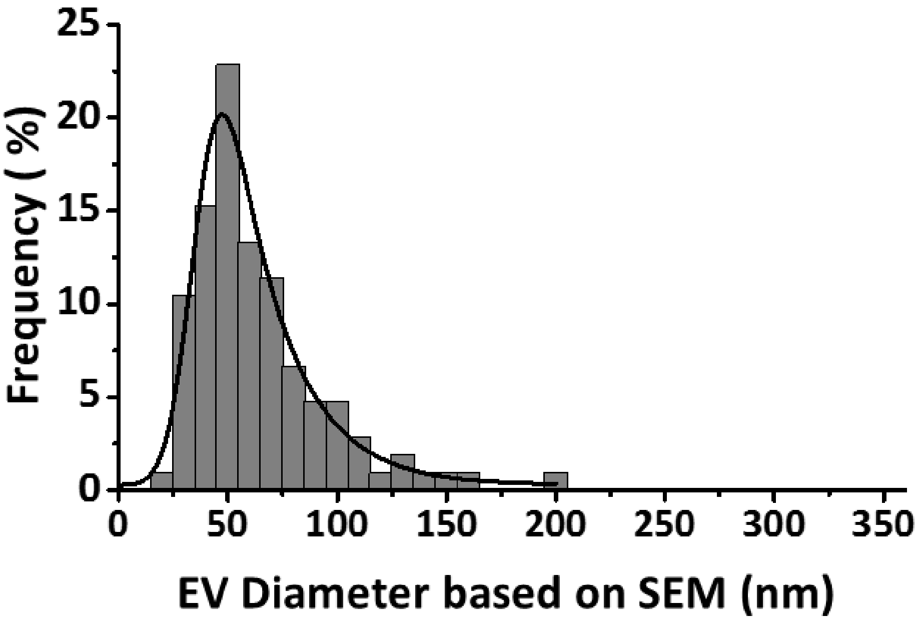

Figure S2. Size distribution of A673 cell-derived EVs on Click Beads based on SEM imaging. n=200, 30-200 nm in diameter.

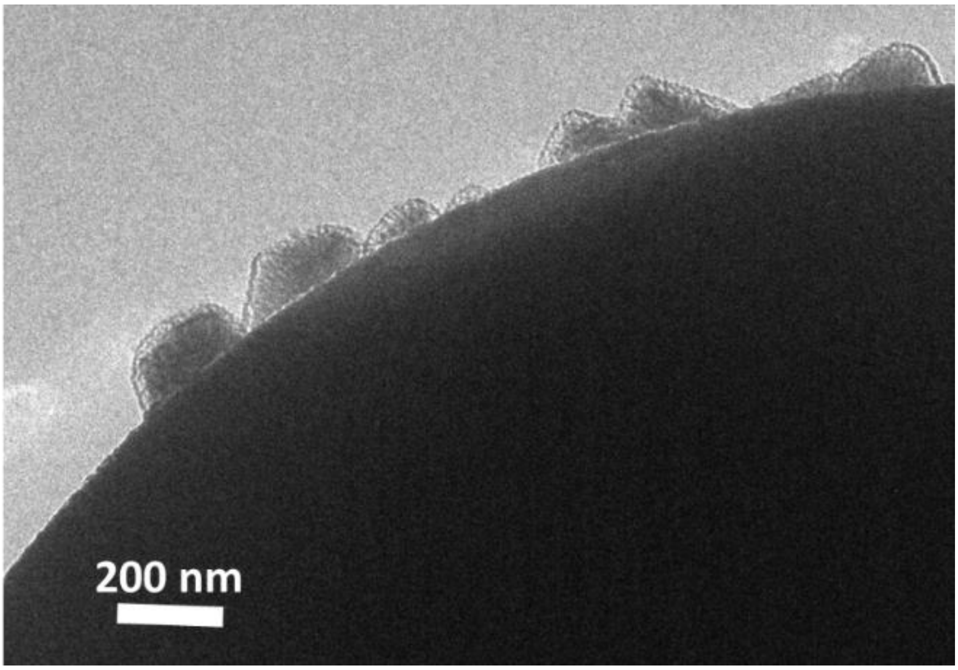

Figure S3. A representative TEM image of EVs captured on a Click Bead.

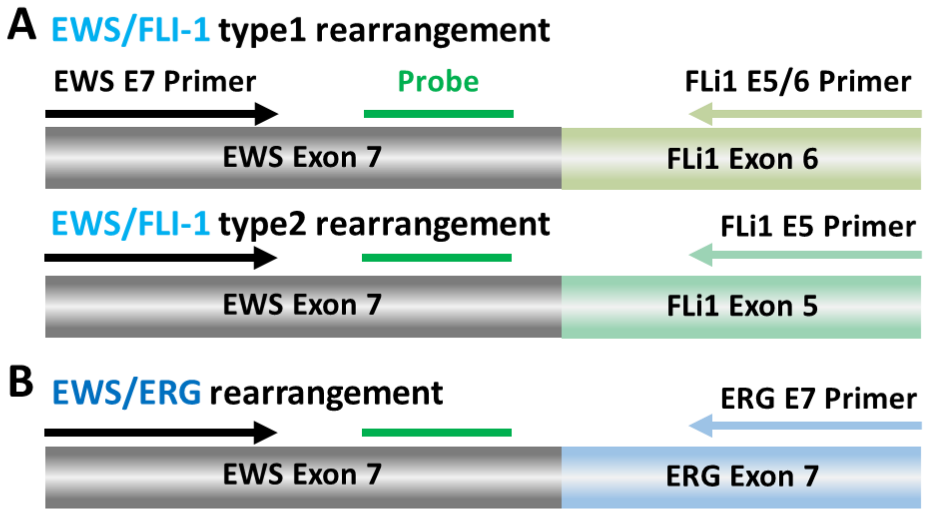

Figure S4. Schematic diagram of EWS rearrangements (EWS/FLI-1 and EWS/ERG).

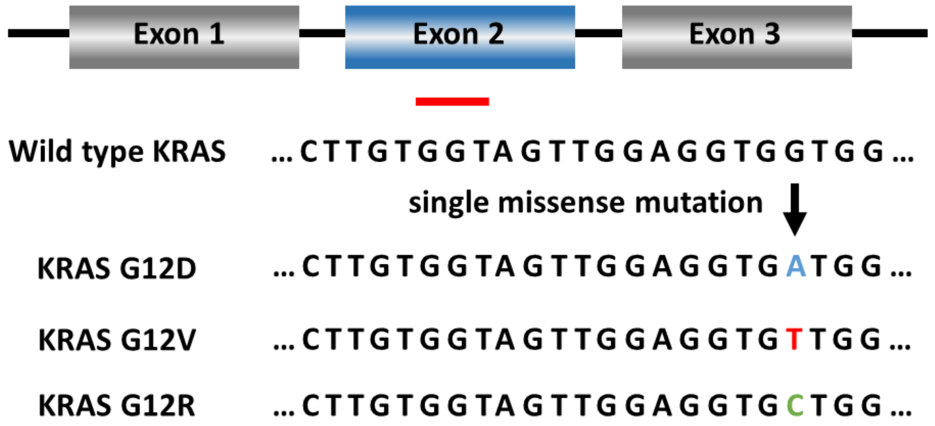

Figure S5. Schematic diagram of KRAS mutations (G12D, G12V and G12R).

Table S1. The atomic concentration (relative) obtained from the integration of high-resolution spectrum after Shirley background subtraction.

Samples	Si	N	C	O	N/Si ratio
**Copper paste**	0.98%	0.00%	80.94%	18.08%	0
**Silica microbeads**	25.07%	0.52%	10.59%	63.82%	0.02
**NH_2-modified microbeads**	24.97%	1.86%	18.66%	54.51%	0.07
**Click Beads**	15.32%	3.74%	39.74%	41.21%	0.24

Table S2. Primers and probes for the detection of EWS rearrangements.

**EWS rearrangements**	**Primers /probes**	**DNA sequence (5’→3’)**
EWS/FLI-1 type 1	Forward	CTA CAG CCA AGC TCC AAG TC
	Reverse	GAC TGA GTC ATA AGA AGG GTT CTG
	Probe	TAG CCA ACA GAG CAG CAG
EWS/FLI-1 type 2	Forward	CTA CAG CCA AGC TCC AAG TC
	Reverse	GTG AGG ATT GGT CGG TGT G
	Probe	TAG CCA ACA GAG CAG CAG
EWS/ERG	Forward	CTA CAG CCA AGC TCC AAG TC
	Reverse	ACC GGT CCA GGC TGA T
	Probe	TAG CCA ACA GAG CAG CAG

Table S3. Clinical characteristics and plasma EV-RNA based detection of EWS rearrangements of sarcoma patients

Patient ID	Gender	Age	Clinical stage	FISH test on tissue	1st blood drawing timepoint	Plasma EV-RNA test			Tissue
						Blood draw	EWS rearrangement	Plasma	
**ES01**	Male	19	IV	EWS +	Treatment naïve	1	45	EWS/FLI-1	EWS/FLI-1
**ES02**	Male	12	IV	EWS +	Under salvage treatment	1	135	EWS/FLI-1	EWS/FLI-1
**ES03**	Male	10 months	IV	EWS +	Treatment naïve	1	120	EWS/FLI-1	NA
**ES04**	Male	18	IV	EWS +	Under salvage treatment	1	26	EWS/FLI-1	NA
						2	79	EWS/FLI-1	
**ES05**	Female	12		EWS +	Post-treatment	1	79	EWS/FLI-1	EWS/FLI-1
**ES06**	Male	12	IV	EWS +	Under salvage treatment	1	23	EWS/FLI-1	NA
**ES07**	Female	8	IV	EWS +	Under salvage treatment	1	113	EWS/FLI-1	NA
						2	15	EWS/FLI-1	
						3	169	EWS/FLI-1	
						4	68	EWS/FLI-1	
**ES08**	Male	20	IV	EWS +	Under salvage treatment	1	173	EWS/FLI-1	NA
						2	319	EWS/FLI-1	
						3	708	EWS/FLI-1	
**ES09**	Female	13	IV	EWS +	Under salvage treatment	1	49	EWS/FLI-1	EWS/FLI-1
						2	60	EWS/FLI-1	
**ES010**	Male	13	IV	EWS +	Treatment naïve	1	75	EWS/FLI-1	NA
**ES011**	Male	23	IV	EWS +	Post-treatment	1	49	EWS/FLI-1	EWS/FLI-1
**ES012**	Female	42	IIIB	EWS +	Post-treatment	1	106	EWS/FLI-1	EWS/FLI-1
**ES013**	Male	46	IB	/	Post-treatment	1	20	EWS/FLI-1	NA
**ES014**	Female	15	IV	EWS +	Post-treatment	1	179	EWS/FLI-1	NA
**ES015**	Male	40	IV	EWS +	Treatment naïve	1	112	EWS/FLI-1	EWS/FLI-1
**ES016**	Female	20	IB	/	Post-treatment	1	325	EWS/FLI-1	NA
**ES017**	Male	32	IV	/	Treatment naïve	1	41	EWS/FLI-1	NA
**ES018**	Female	17	IV	EWS +	Post-treatment	1	33	EWS/FLI-1	EWS/FLI-1
**ES019**	Male	23	IV	EWS +	Post-treatment	1	64	EWS/FLI-1	NA
**ES020**	Female	55	IA	EWS +	Treatment naïve	1	97	EWS/FLI-1	EWS/FLI-1
**ES021**	Male	51	IA	/	Post-treatment	1	355	EWS/FLI-1	NA
**ES022**	Male	18	IV	EWS +	Treatment naïve	1	56	EWS/FLI-1	EWS/FLI-1
**ES023**	Male	14	IV	EWS +	Treatment naïve	1	19	EWS/FLI-1	NA
**ES024**	Female	37	IV	EWS +	Treatment naïve	1	521	EWS/FLI-1	NA
**ES025**	Female	27	IV	EWS +	Post-treatment	1	357	EWS/FLI-1	EWS/FLI-1
**ES026**	Male	13	IIIA	EWS +	Treatment naïve	1	83	EWS/FLI-1	NA
**ES027**	Male	67	IV	/	Post-treatment	1	300	EWS/FLI-1	NA
**ES028**	Female	17	IV	EWS +	Post-treatment	1	34	EWS/FLI-1	EWS/FLI-1

Table S4. Clinical characteristics and plasma EV-RNA based detection of healthy donors

HD	Gender	Age	Plasma EV-RNA test					
			EWS/FLI-1	EWS/ERG	G12D	G12V	G12R	WT
**HD1**	Female	40	0	0	0	0	0	3413
**HD2**	Female	28	0	0	0	0	0	13056
**HD3**	Female	34	0	0	0	0	0	4455
**HD4**	Male	60	0	0	0	0	0	2488
**HD5**	Male	23	0	0	0	0	0	16317
**HD6**	Male	57	0	0	0	0	0	5557
**HD7**	Male	50	0	0	0	0	0	8631
**HD8**	Male	26	0	0	0	0	0	6845
**HD9**	Male	44	0	0	0	0	0	393
**HD10**	Female	57	0	0	0	0	0	4500

Table S5. Primers and probes for the detection of KRAS mutations.

**KRAS mutations**	**Assay ID**	
G12D	Hs000000050_rm	Catalog #A44177
G12V	Hs000000051_rm	Catalog # A44177
G12R	Hs000000049_rm	Catalog # A44177

Table S6. Clinical characteristics and plasma EV-RNA based detection of KRAS mutation of pancreatic patients

Patient ID	Gender	Age	1st blood drawing timepoint	blood draw	CA 19-9	Plasma EV-RNA test		Tissue
						KRAS transcripts	Mutation	
**PanC01**	Male	56	Treatment naïve	1	215	6700	G12D	G12D
				2	411	22935		
				3	640	23947		
				4	780	340963		
				5	974	62761		
**PanC02**	Female	67	Under treatment	1	2243	1125	G12V	G12V
				2	222572	21855		
				3	1629800	235185		
**PanC03**	Male	79	Treatment naïve	1	708	310259	G12D	NA
				2	259	37500		
				3	NA	5875		
				4	65	10308		
				5	38	13973		
				6	70	45867		
				7	101	19004		
**PanC04**	Male	74	Under treatment	1	1320	8208	G12V	G12V
				2	6381	363079		
**PanC05**	Female	76	Under treatment	1	18068	6105	G12D	G12D
				2	19384	23623		
**PanC06**	Male	65	Under treatment	1	13396	17419	G12R	G12R
				2	33175	51447		
**PanC07**	Male	83	Treatment naïve	1	69	38292	G12R	WT
**PanC08**	Female	77	Treatment naïve	1	3	31052	WT	WT
**PanC09**	Male	96	Treatment naïve	1	14375	13176	G12D	NA
				2	NA	39983		
**PanC10**	Female	68	Treatment naïve	1	12	19403	WT	WT
**PanC11**	Female	70	Treatment naïve	1	NA	6483	WT	NA
**PanC12**	Female	69	Under treatment	1	39	11833	WT	NA
**PanC13**	Female	75	Under treatment	1	23	9887	G12D	WT
**PanC14**	Male	68	Treatment naïve	1	NA	14762	WT	NA
**PanC15**	Male	69	Treatment naïve	1	NA	950542	G12D	G12D
**PanC16**	Female	65	Treatment naïve	1	321	13556	G12D	G12D
**PanC17**	Male	74	Under treatment	1	181	7536	WT	WT
**PanC18**	Male	67	Under treatment	1	44	25875	G12D	NA
**PanC19**	Male	77	Treatment naïve	1	8	24792	WT	G12D
